# Selenite Cystine Agar for Enumeration of Inoculated *Salmonella* Serovars Recovered from Stressful Conditions during Antimicrobial Validation Studies

**DOI:** 10.3390/microorganisms8030338

**Published:** 2020-02-28

**Authors:** Caitlin E. Karolenko, Arjun Bhusal, Dhiraj Gautam, Peter M. Muriana

**Affiliations:** 1 Robert M. Kerr Food & Agricultural Products Center, Oklahoma State University, Stillwater, OK 74078, USA; caitlin.e.karolenko@okstate.edu (C.E.K.); arjun.bhusal@okstate.edu (A.B.); 2 Department of Animal and Food Sciences, Oklahoma State University, Stillwater, OK 74078, USA; 3 Los Angeles County, Department of Agricultural Commissioner/Weights & Measures, South Gate, CA 90280, USA; dhirajgm@gmail.com

**Keywords:** *Salmonella*, acid adaptation, stress, Xylose Lysine Desoxycholate, Hektoen Enteric, Selenite Cystine, antibiotics, inoculum

## Abstract

Process validation studies often require the inoculation of select foodborne pathogens into targeted foods to determine the lethality of the process or antimicrobial ingredients, and quantitative recovery of surviving inoculum bacteria helps to make those assessments. Such processes introduce various stressors on the inoculated challenge microorganisms whereby traditional selective media are too harsh to enumerate the remaining viable and injured population quantitatively. Innate antibiotic resistance of challenge organisms has often been used to establish simple selective media (i.e., Tryptic Soy Agar/TSA + antibiotics) for recovering inoculated strains, but sometimes antibiotic resistant background microorganisms are higher than desired. *Salmonella* Thompson 120, *Salmonella* Heidelberg F5038BG1, *Salmonella* Hadar MF60404, *Salmonella* Enteritidis H3527, and *Salmonella* Typhimurium H3380 were characterized for antibiotic resistance and acid adaptation in Tryptic Soy Broth containing 0%, 0.25%, or 1.0% glucose. Sodium pyruvate was evaluated for recovery after stress but no enhancing effect was observed, possibly because the strains were acid-adapted. Selenite Cystine Broth, traditionally used as a selective enrichment broth, was used as the basis for Selenite Cystine Agar (SCA) in combination with three antibiotics to which our *Salmonella* are resistant. Serovars of *Salmonella*, both individually and in mixtures, were enumerated on TSA, SCA, Xylose Lysine Desoxycholate (XLD), and Hektoen Enteric (HE) selective agars (all containing the same antibiotics) after conditions of nutrient starvation, desiccation, acid stress, and thermal stress. The data show that quantitative enumeration of our *Salmonella* serovars on SCA was not significantly different (*p* > 0.05) than those achieved on TSA for all tested stress categories. Levels of *Salmonella* enumerated on XLD and/or HE were significantly different (*p* < 0.05) than on TSA and SCA and often more than 1–2-log lower, consistent with the inhibition of injured cells. These data confirm that SCA (+ antibiotics) is a suitable selective medium for enumeration of these acid-adapted *Salmonella* serovars as challenge organisms recovered from various conditions of stress.

## 1. Introduction

Microbial challenge studies of foods are often conducted using pathogens or spoilage microorganisms that are inoculated into targeted food products. After processing or some period of shelf life, the inoculated microorganisms are recovered and enumerated to determine whether the food formulation or food processing conditions inhibits (bactericidal), prevents growth (bacteriostatic), or allows survival and growth (no control) under the conditions of treatment. Such inoculates are often added to products that are not sterile and may include a background of other microorganisms from which the inoculum must be differentially enumerated. Sometimes, no selective media is required for the inoculated microorganisms if the level of inoculum is significantly higher (several orders of magnitude) than the underlying background. Enumeration of un-inoculated control samples provides proof that the background microbiota are well below the inoculum level in test samples.

The use of simple media containing one or more antibiotics for which the added strains are resistant can readily be employed to provide sufficient ‘knock down’ of background organisms. Antibiotic resistance can be generated by the selective recovery of low frequency (10^-6^) spontaneous mutations incurred during selective pressure of high cell levels plated on antibiotic containing media [1]. These mutational changes in DNA can eliminate target binding sites of antibiotics that normally bind to RNA polymerase (rifampin, rifamycin) or ribosomes (streptomycin, spectinomycin, gentamycin) affecting transcription or translation, respectively, and provide stable resistance to those antibiotics [2,3]. Numerous examples using this basic approach are found in the published literature [4,5,6,7,8], and is supported by the National Advisory Committee for the Microbial Criteria of Food (NACMCF) [9] as a method of selective recovery of inoculated strains from foods.

More elaborate approaches have examined whether selective/differential media used for ‘detection’ of foodborne pathogens may also be adapted for purposes of microbial ‘enumeration’. The use of such agars for differential enumeration has often lead to inaccurate underreporting of pathogenic populations because such media are harsh on injured cells and may result in significantly lower microbial counts [10,11]. Accurate enumeration can be enhanced by a variety of substances that may improve recovery of injured bacteria from stressed conditions. Sodium pyruvate, yeast extract, free radical scavengers (superoxide dismutase, catalase), cations (zinc), or diluent/media buffers may improve recovery of injured bacteria after stressed conditions [12,13,14,15,16,17]. In addition to media additives, various layered agar methods (i.e., direct overlay, thin agar overlay, and agar underlay method) have also been used to improve recovery of injured cells that might otherwise be suppressed by selective media [18,19,20,21].

The current work describes our efforts to characterize several selective media for enumeration of widely used *Salmonella* serovars that are often added as challenge inocula in process validation studies and recovered after exposure to acidic antimicrobial treatments, nutrient depletion, thermal treatment, or desiccation.

## 2. Materials and Methods

### 2.1. Bacterial Strains and Growth Conditions

Active cultures were grown in Tryptic Soy Broth (TSB, BD Bacto, Franklin Lakes, NJ, USA) in 9-mL tubes at 37°C. Cultures were maintained for storage by centrifugation (6,000xg, 5°C) of 9 mL of fresh, overnight cultures and cell pellets were resuspended in 2–3 mL of fresh sterile TSB containing 10% glycerol. Cell suspensions were placed into glass vials and stored in an ultra-low freezer (−80°C). Frozen stocks were revived by transferring 100 µL of the thawed cell suspension into 9 mL of TSB, incubating overnight at 37°C, and sub-cultured twice before use. Microbial enumeration for all assays was carried out on Tryptic Soy Agar (TSA, BD Bacto; 1.5% agar) and plated in duplicate.

*Salmonella* serovars used in this study included: *Salmonella enterica subsp. enterica* serotype Thompson 120 (chicken isolate), *Salmonella enterica subsp. enterica* serotype Heidelberg F5038BG1 (ham isolate), *Salmonella enterica subsp. enterica* serotype Hadar MF60404 (turkey isolate), *Salmonella enterica subsp. enterica* serotype Enteritidis H3527 (phage type 13a, clinical isolate), *Salmonella enterica subsp. enterica* serotype Typhimurium H3380 (DT 104 clinical isolate), and *Salmonella enterica subsp. enterica* serotype Montevideo FSIS 051 (beef isolate). These are well-characterized strains that have been used in numerous research publications involving antimicrobial interventions against *Salmonella* spp [22,23,24,25].

Acid adaptation of our *Salmonella* serovars was carried out according to Wilde et al. [26] in which cultures were inoculated in TSB augmented with 1% glucose prior to use [27]. Individual cultures were harvested by centrifugation, and resuspended with 0.1% buffered peptone water (BPW, BD Difco) and held refrigerated until use (5 °C). In situations where a mixed-inoculum was used, the centrifuged and resuspended individual cultures were mixed in equal proportions. All stress tests in this study were performed using acid-adapted *Salmonella* cultures in TSA containing 1% glucose as described above. The US Department of Agriculture, Food Safety Inspection Service (USDA-FSIS) ‘highly recommends’ the use of acid-adapted cultures when such inoculum strains would be used for stressed conditions to ensure that they are not easily overcome by acidic processing conditions.

Confirmation of pH effects of *Salmonella* grown in media containing glucose was examined in three different TSB media: TSB containing 0% glucose (BD Bacto, BD286220), 0.25% glucose (BD Bacto, BD211825), and 1% glucose (BD286220 + 1% glucose). All cultures were separately-inoculated into TSB media containing 0% glucose (in triplicate replication) and incubated overnight at 37 °C; these cultures in turn, were used to inoculate different replicative sets of TSB at 0%, 0.25%, and 1.0% glucose and pH levels of the various cultures were then recorded after 18 hrs at 37 °C.

### 2.2. Antibiotics, Disc Assay, and Media Validation of Antibiotic Resistance

Five *Salmonella* serovars were tested for innate antibiotic resistance using BD BBL Sensi-Discs (Becton-Dickenson Laboratories, Franklin Lakes, NJ, USA) consisting of sterile paper discs impregnated with specific levels of antibiotic [28]. Bacterial lawns were obtained for individual *Salmonella* serovars by seeding 0.1 mL of overnight culture into 10 mL molten/tempered TSA (0.75% agar), mixed, and overlaid onto pre-poured TSA (1.5% agar) in 150-mm petri plates. When the overlay was solidified, antibiotic discs were aseptically dispensed onto the bacterial lawns and plates were incubated overnight at 37 °C. Following incubation, cultures were evaluated for resistance (no zone) or degree of susceptibility based on subjective size of the inhibitory zone (slightly sensitive, sensitive, very sensitive).

Antibiotics examined included Amikacin (30 ug), Ampicillin (10 ug), Cefazolin (30 ug), Cefotaxime (30 ug), Cefoxitin (30 ug), Cephalothin (30 ug), Chloramphenicol (30 ug), Chloramphenicol (5 ug), Ciprofloxacin (5 ug), Clindamycin (2 ug), Colistin (10 ug), Erythromycin (15 ug), Ethionamide (25 ug), Furazolidone (100 ug), Gentamicin (10 ug), Isoniazid (5 ug), Nalidixic acid (30 ug), Nitrofurantoin (300 ug), Novobiocin (5 ug), Oxacillin (1 ug), Penicillin (10 units), Piperacillin (100 ug), Rifampin (5 ug), Streptomycin (10 ug), Streptomycin (50 ug), Tetracycline (30 ug), Tobramycin (10 ug), Vancomycin (30 ug) (BD Labs).

Antibiotic resistance was confirmed on agar by plating individual cultures grown in TSB (without antibiotics) for comparative enumeration onto TSA plates, with and without individual antibiotics. This was especially important for combinations of antibiotics to insure the absence of synergistic inhibitory activity when multiple antibiotics are combined. All assays were performed in triplicate replication.

### 2.3. Salmonella-Selective Agar Media Used for Enumeration of Salmonella spp. after Stressed Conditions

Four selective agar media were compared for enumeration of acid-adapted *Salmonella* serovars after various stress situations including nutrient depletion, acid stress, desiccation, and thermal stress to mimic conditions from which they may be recovered when examining various antimicrobial/processing conditions. The selective media included TSA (non-selective), Selenite Cystine Agar (SCA), Hektoen Enteric (HE), and Xylose Lysine Desoxycholate (XLD) agars. All four of these agar media contained three antibiotics: Spectinomycin (5 ug/mL), clindamycin (5 ug/mL), and novobiocin (50 ug/mL). Each of the individual *Salmonella* serovars were retrieved after consecutive passage (streak isolation) from one agar media to the other to insure clonal isolates would be tolerant of each media before growth and storage of cultures as frozen stocks.

### 2.4. Preliminary Studies: Evaluation of Sodium Pyruvate for Recovery of Injured Bacterial Cells

In addition to various selective media and antibiotics to which the *Salmonella* strains were resistant, sodium pyruvate (0.1%) was evaluated to determine if it enhanced the recovery of injured *Salmonella.* Optimal levels of sodium pyruvate for recovery of stressed/injured cells have been reported as low as 0.05% to as high as 1.0% [29,30,31].

#### 2.4.1. Sodium Pyruvate Following Acid Adaptation and Nutrient Starvation

Acid-adapted cultures grown in TSB (1% glucose) were centrifuged and resuspended in a reduced volume of 0.1% BPW to concentrate cells approximately 10-fold and combined in equal amounts. The mixed culture suspension was further diluted in a 10-fold dilution series with 0.1% BPW and maintained at 4 °C for 10 days to induce starvation, as per Wesche et al. [32] and Dickson and Frank [33]. The stored dilutions were then plated on TSA, SCA, XLD, and HE and on the same agars containing 0.1% sodium pyruvate (i.e., TSA-SP, SCA-SP, XLD-SP, HE-SP), and incubated at 37 °C for 48 hrs before enumeration. Each of the agar media contained spectinomycin (5 ug/mL), novobiocin (50 ug/mL), and clindamycin (5 ug/mL).

#### 2.4.2. Sodium Pyruvate Following Acid Adaptation, Salt Desiccation, and Acid Exposure

Mixed acid-adapted *Salmonella* serovars, as described above, were surface-inoculated (150 uL/side) onto ~100 gm beef pieces, vacuum-marinated (15 inches Hg) for 30 min in a vacuum tumbler (Biro VTS-43, Marblehead, OH, USA) in a biltong spice blend containing 2% salt and 3% vinegar (10% acetic acid) (% of ingredient is listed as a % of total formulation). Following marination, beef samples were stomached with 100 mL of neutralizing buffered peptone water (nBPW; Hardy Diagnostics, Santa Maria, CA, USA) from which additional 10-fold dilutions were made in 0.1% BPW. These dilutions were then plated on the same selective plates described above (with and without sodium pyruvate; all containing three antibiotics) and incubated at 37 °C for 48 hrs before enumeration.

### 2.5. Stress Conditions for Enumeration

Multiple selective agar media (TSA, SCA, XLD, and HE) containing spectinomycin (5 ug/mL), clindamycin (5 ug/mL), and novobiocin (50 ug/mL) were compared for enumeration of *Salmonella*, either individually or in mixture, under different stress conditions including: nutrient depletion, acid stress, desiccation, and thermal treatment.

#### 2.5.1. Nutrient Depletion and Cell Starvation

**Figure 1:**
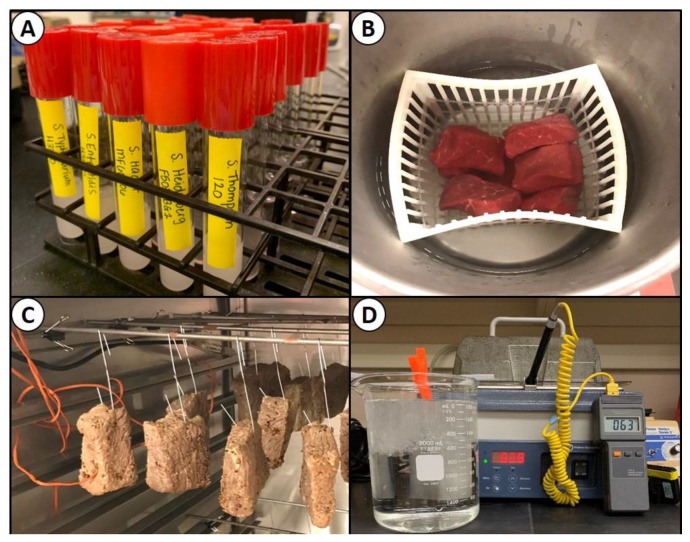
Stress conditions for *Salmonella* serovars: (**A**) nutrient depletion/starvation by extended refrigeration of individual *Salmonella* serovars in 0.1% BPW; (**B**) acid stress by dip treatment of *Salmonella*-inoculated beef in acidic solutions (vinegar, 5%; 3% sodium acid sulfate; 5% lactic acid); (**C**) desiccation of *Salmonella*-inoculated and spice-coated beef at 23.9 °C (75 °F) and 55% RH in a temperature-controlled humidity oven; (**D**) thermal heating of vacuum-packaged bags containing *Salmonella* inocula at 62.8 °C (145 °F) for 75 sec.

Each of five individual *Salmonella* serovars, in triplicate replication, were grown in TSB containing 1% glucose, serially diluted in 0.1% BPW, and surface plated immediately after harvesting; at this point, these were referred to as ‘fresh cells’. The *Salmonella* were plated onto four selective media (TSA, SCA, HE, and XLD), each containing three antibiotics: Spectinomycin (5 ug/mL), clindamycin (5 ug/mL), novobiocin (50 ug/mL). Plates were then incubated at 37 °C for 48 h. Nutrient starvation was assessed by maintaining the dilution tubes used for the fresh cell experiment at 4 °C and determining the bacterial populations after extended three- and six-week intervals (Figure 1A). The trials were performed in triplicate, with each dilution plated in duplicate, on TSA, SCA, HE, and XLD (all containing three antibiotics), and incubated for 48 h at 37 °C.

#### 2.5.2. Antimicrobial (Acid) Stress

Intact, select grade, beef bottom-round sub-primal cuts (Ralph’s Packing Co., Perkins, OK, USA) were trimmed to approximately 0.75-in thick × 2-inch wide × 3-inch long ‘steaks’ at the R.M. Kerr Food & Agricultural Product Center (FAPC). Beef pieces used for this experiment were vacuum-packaged fresh, stored frozen (−20 °C), and thawed immediately before use. The beef pieces were inoculated by pipette with 150 uL of the 5-serovar *Salmonella* mixture on each side, and immediately spread with a ‘gloved finger’. Inoculated beef pieces were then incubated for 30 min at 4–5 °C to allow for bacterial attachment prior to use. Following incubation, the inoculated beef was dipped in white vinegar (5% acetic acid), lactic acid (5%), or sodium acid sulfate (SAS, 3%) for 30 s and excess liquid was allowed to drain before proceeding (Figure 1B). Meat samples were transferred to filter-stomaching bags, followed by the addition of 100 mL of 1% nBPW (Hardy Diagnostics) and then stomached for 90 sec in a Masticator paddle-blender (IUL Instruments, Barcelona, Spain). Samples were withdrawn, serially-diluted with 0.1% BPW, and dilutions surface plated (0.1 mL) in duplicate, on TSA, SCA, HE, and XLD plates (each containing spectinomycin, clindamycin, and novobiocin) and incubated at 37 °C for 48 h before enumeration. All trials were performed as separate experiments in triplicate replication.

#### 2.5.3. Desiccation and Drying

Beef pieces, as described previously, were inoculated with 150 uL of a five-serovar *Salmonella* mixture on each side that was spread with a gloved finger and held at 4–5 °C for 30 min to allow for bacterial attachment. The beef pieces were then tumbled (without vacuum) in a biltong spice mixture (2% salt) for 5 min until pieces were evenly coated. Beef pieces were then hung in a temperature-controlled humidity oven (Hotpack, Warminster, PA, USA) at 23.9 °C (75 °F) and 55% relative humidity (RH) and allowed to dry for up to 4 days (Figure 1C). Beef was sampled after inoculation (0 days), and after 2 and 4 days of drying. Beef samples were stomached with 1% nBPW and then serially diluted in 0.1% BPW. Serial dilutions were plated in duplicate (0.1 mL) on the surface of TSA, SCA, HE, and XLD (each containing spectinomycin, clindamycin, and novobiocin) and incubated for 48 h at 37 °C. Treatments were performed in triplicate replication.

#### 2.5.4. Thermal Stress

*Salmonella* serovar cultures were grown in TSB (+ 1% glucose) as described earlier, harvested by centrifugation, resuspended in fresh/sterile TSB (+ 1% glucose), mixed in equal volumes, and held on ice until used. Then, 1.0 mL of the mixed culture was heat-sealed as a thin layer in vacuum-package bags and heated at 145 °F (62.8 °C) for 75 sec (Figure 1D). The cultures were then removed to ice water to chill for 15 min and held at room temperature for 5 min. Dilutions were then made in 0.1% BPW and surface plated (0.1 mL) in duplicate onto TSA, SCA, HE, and XLD agars (each containing spectinomycin, clindamycin, and novobiocin) and incubated for 48 h at 37 °C. All treatments, including inoculations, were performed in triplicate replication.

### 2.6. Bacterial Injury

The degree of bacterial sublethal injury was determined by comparing microbial counts on nonselective media (TSA) to those on selective media (XLD) for various treatments in the prior trials according to the equation described by Wesche et al. [32]:

(1) $$\%\;Injury=\frac{\left[\left(count\;on\;nonselective\;agar\right)-\left(count\;on\;selective\;agar\right)\right]}{\left(count\;on\;nonselective\;agar\right)}\;x\;100$$

### 2.7. Statistical Analysis

Each trial was performed in triplicate replication and all replications were performed as autonomous and separate experiments using separately inoculated cultures and prepared plating media. All data were presented as the mean of triplicate replications with standard deviation of the mean represented by error bars. Statistical analysis was done using one-way analysis of variance (ANOVA) and the Holm–Sidak test for pairwise multiple comparisons to determine significant differences (*p* < 0.05). Data treatments with different letters are significantly different (*p* < 0.05); treatments with the same letter are not significantly different (*p* > 0.05).

## 3. Results

### 3.1. Acid Adaptation of Salmonella Cultures

**Figure 2:**
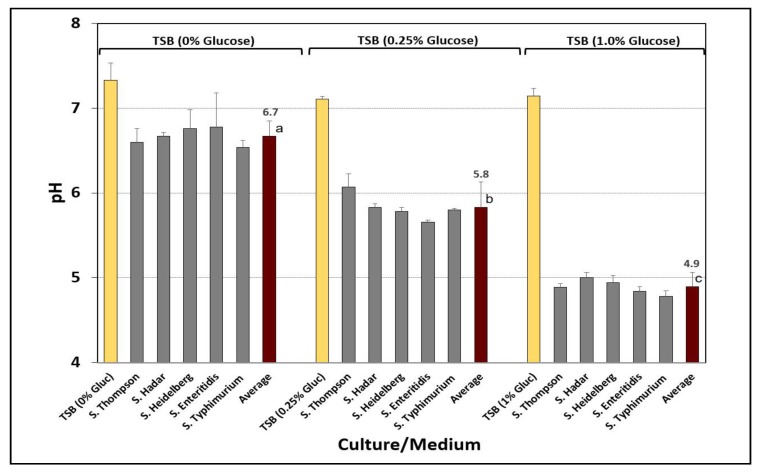
Analysis of spent broth pH for *Salmonella* cultures grown in Tryptic Soy Broth (TSB) containing 0%, 0.25%, or 1% glucose incubated at 37 °C for 18 hrs. The data bars in each set are pH values for the medium before inoculation, the five individual cultures after growth, and the average pH of the five cultures. Cultures include *S.* Thompson 120, *S.* Heidelberg F5038BG1, *S.* Hadar MF60404, *S.* Enteritidis H3527, and *S.* Typhimurium H3380. Data are presented as the mean of triplicate replications and error bars represent the standard deviation from the mean. Means (for average pH) with different letters are significantly different, as determined by one-way ANOVA using the Holm–Sidak test for pairwise multiple comparisons to determine significant differences (*p* < 0.05); means with different letters are significantly different (*p* < 0.05).

Cultures were grown in TSB containing 1% glucose in order to ‘acid adapt’ them to low pH for all stress conditions used in this study. The pH of each of five different *Salmonella* serovar cultures was examined after growth in TSB, with and without glucose (0%, 0.25%, and 1.0% glucose). Growth in TSB without glucose resulted in spent culture broth pH near neutrality (i.e., average, pH 6.7) whereas the culture broth pH of those grown in TSB with glucose were significantly lower (i.e., 0.25% glucose, average, pH 5.8; 1.0% glucose, average, pH 4.9) (Figure 2).

### 3.2. Determination of Salmonella Antibiotic Resistance (Disc Assay)

Antibiotic resistance for six *Salmonella* serovars was examined using BD BBL Sensi-Discs on lawns of individual *Salmonella* serovars and scored qualitatively for sensitivity or resistance to the antibiotic discs (Table 1; only those antibiotics for which four or more strains were resistant are shown). ‘Resistance’ was characterized as an antibiotic disc that showed no inhibition zone around the periphery of the disc while various degrees of sensitivity were subjectively attributed according to size of a visible inhibition zone.

### 3.3. Confirmation of Salmonella Antibiotic Resistance (Plating on Agar Containing Antibiotics)

**Figure 3:**
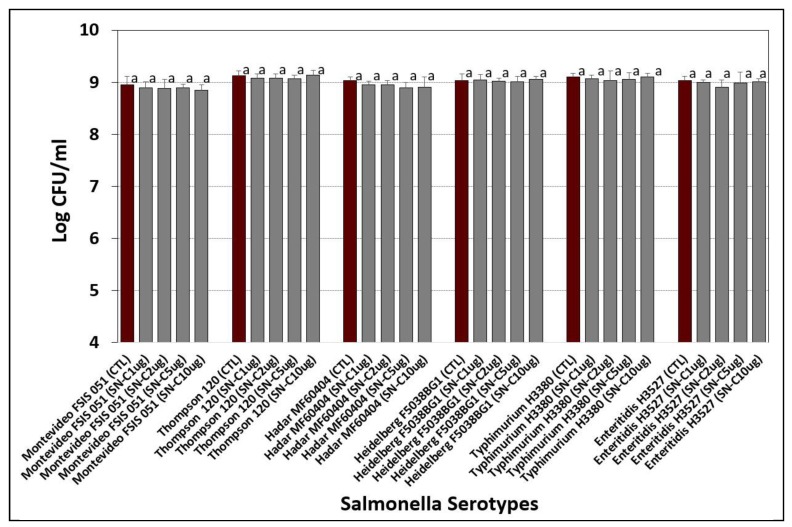
Evaluation of *Salmonella* serovars on TSA alone as Control (CTL) vs. TSA containing spectinomycin (S, 10 ug/mL), novobiocin (N, 100 ug/mL), and increasing amounts of clindamycin (C, 1-, 2-, 5-, and 10 ug/mL). Cultures include *S.* Montevideo FSIS 051, *S.* Thompson 120, *S.* Heidelberg F5038BG1, *S.* Hadar MF60404, *S.* Enteritidis H3527, and *S.* Typhimurium H3380. Cultures were grown 18–20 hrs at 37 °C in TSB (without antibiotics), 10-fold dilutions in 0.1% BPW, and plated on the various media as indicated. Data are presented as the mean of triplicate replications and error bars represent the standard deviation from the mean. Within the same serotype, comparisons of means with the same letters are not significantly different (*p* > 0.05) as determined by one-way ANOVA using the Holm–Sidak test for pairwise multiple comparisons to determine significant differences.

Based on the antibiotic disc assay (Table 1), ‘on agar’ antibiotic resistance was confirmed by growing cultures in TSB (without antibiotics) and plating the cultures on TSA alone, and on TSA containing the chosen antibiotics (Figure 3). This was done to determine if there was any antagonism that might be at play when multiple antibiotics are added in combination [34,35,36,37].

### 3.4. Selective Agar Media Containing Antibiotics

Based on the microbial platings of the individual *Salmonella* serovars on TSA containing spectinomycin, novobiocin, and clindamycin at 10-, 100-, and 10-ug/mL, respectively, we felt these levels, or even slightly lower in TSA or other media, would provide a good ‘selective medium’ for our specific *Salmonella* serovar inoculum in various applications with raw meat if we could demonstrate enumeration equivalent to that on TSA. Additional platings with HE and XLD agars, both with and without antibiotics, showed no significant differences (*p* > 0.05) (data not shown).

#### Tryptic Soy Agar and Selenite Cystine Agar Containing Antibiotics

**Figure 4:**
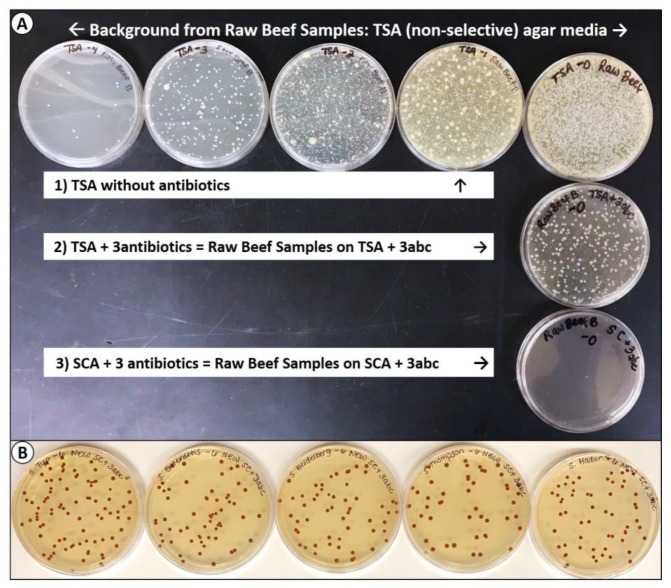
Comparison of TSA (without antibiotics) vs. TSA and selinite cystine agar (SCA) (with antibiotics). (**A**) Plating of samples taken from fresh raw meat onto TSA without antibiotics (top row), vs. TSA containing antibiotics (2nd row), vs. SCA containing antibiotics (3rd row). The “3abc” represents the ‘three antibiotics’ described previously: Spectinomycin (5 ug/mL), novobiocin (50 ug/mL), and clindamycin (5 ug/mL). (**B**) Individual *Salmonella* serovars surface plated (10^−6^ dilution) on SCA containing three antibiotics.

TSA containing spectinomycin (5 ug/mL), clindamycin (5 ug/mL), and novobiocin (50 ug/mL) was examined in a variety of situations where multi-strain (serovar) combinations of *Salmonella* were used. However, we obtained very high background counts on some samples of raw meat on TSA without antibiotics (Figure 4A, top row). When plated on TSA containing the three antibiotics (i.e., 3abc), the background levels were reduced by approximately 3 logs, but still had significant levels of colonies on the lowest dilution (Figure 4A, middle row). This background was further minimized when we used SCA in combination with our antibiotics for which our *Salmonella* serovars were resistant (Figure 4A, bottom row). Each of the *Salmonella* serovars gave slightly different colony sizes on TSA + 3abc for the same incubation time (data not shown) whereas they grew luxuriously on the SC agar (SCA) giving equivalent-sized and perfectly round colony morphologies (Figure 4B).

### 3.5. Comparitive Enumeration on Selective Agars with and without Sodium Pyruvate

Selective agars were formulated that consisted of TSA, TSA-SP, SCA, SCA-SP, XLD, XLD-SP, HE, and HE-SP, all containing three antibiotics (spectinomycin, 5 ug/mL; novobiocin, 50 ug/mL; clindamycin, 5 ug/mL). Individual *Salmonella* serovars, or equal mixtures of them, were enumerated on these four media after various stress conditions.

#### Evaluation of Sodium Pyruvate for Recovery of Injured Cells

**Figure 5:**
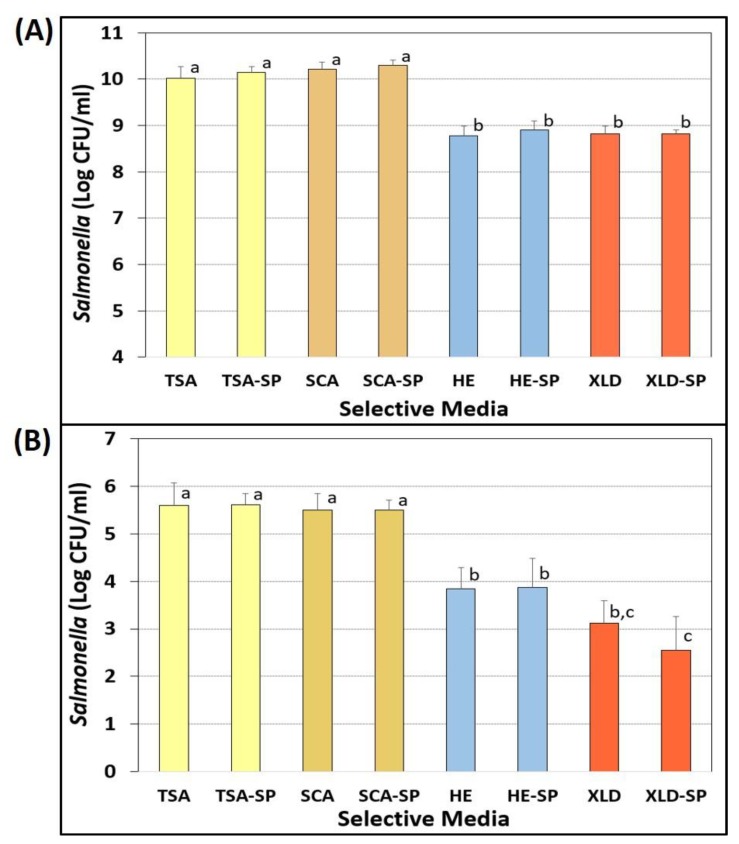
Evaluation of sodium pyruvate for recovery of injured *Salmonella* (**A**) after 10-day nutrient starvation or (**B**) after 30-min salt and vinegar (acid) marinade treatment on inoculated beef pieces. Enumeration of mixed-*Salmonella* serovars on TSA, SCA, Hektoen Enteric (HE), Xylose Lysine Desoxycholate (XLD), and on the same media containing 0.1% sodium pyruvate (TSA-SP, SCA-SP, HE-SP, XLD-SP). All media contained spectinomycin (5 ug/mL), novobiocin (50 ug/mL), and clindamycin (5 ug/mL). Cultures included *S*. Thompson 120, *S*. Heidelberg F5038BG1, *S*. Hadar MF60404, *S*. Enteritidis H3527, and *S*. Typhimurium H3380. Data are presented as the mean of triplicate replications, and error bars represent the standard deviation from the mean. Comparisons of means with different letters are significantly different (*p* < 0.05) as determined by one-way ANOVA using the Holm–Sidak test for pairwise multiple comparisons; means with the same letter are not significantly different (*p* > 0.05).

Sodium pyruvate was examined as a possible supplemental ingredient for the recovery of injured bacterial cells from acid-adapted and nutrient-starved Salmonella (Figure 5A) as well as acid-adapted and 2% salt/3% vinegar-marinaded *Salmonella* (Figure 5B). When nutrient-starved Salmonella were plated on the various selective media, no statistical difference (*p* > 0.05) was observed between TSA, TSA-SP, SCA, and SCA-SP, even though the data for SCA was slightly higher than TSA (Figure 5A). However, microbial counts on both HE and XLD (with and without sodium pyruvate), were approximately 1.5-log lower and significantly different (*p* < 0.05) than TSA and SCA. No significant differences (*p* > 0.05) were observed between HE and HE-SP, nor between XLD and XLD-SP (Figure 5A).

When inoculated beef pieces were subjected to a salt/vinegar spice marinade and vacuum-tumbled for 30 min, we observed lower counts as would be expected from salt/vinegar marination (inhibition) and subsequent sample stomaching of beef in 100 mL buffer (dilution). Results show that no significant difference (*p* < 0.05) was observed among TSA- or SCA-based media (with or without sodium pyruvate), nor between HE and HE-SP or XLD and XLD-SP (Figure 5B). Again, enumerations on HE, HE-SP, XLD, and XLD-SP were significantly different (*p* < 0.05) and lower than on TSA and SCA (Figure 5B).

### 3.6. Salmonella Stress Conditions: Nutrient Depletion and Starvation

**Figure 6:**
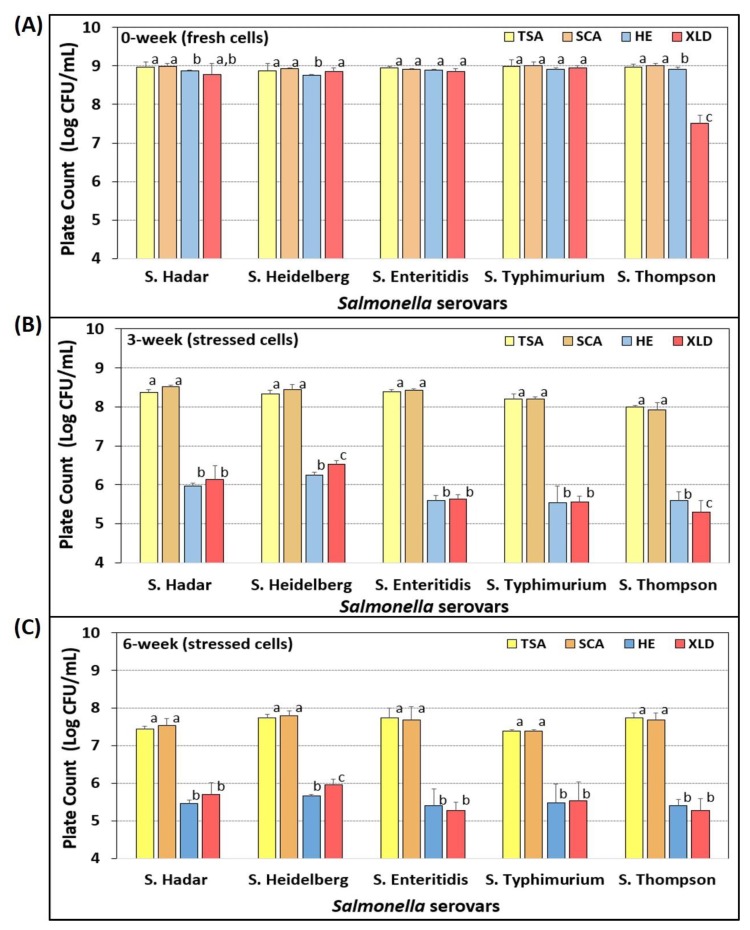
Comparison of four selective media for enumeration of individual *Salmonella* serovars after extended nutrient starvation for 0-weeks (**A**), 3-weeks (**B**), and 6-weeks (**C**) at 4 °C. Each serovar was plated on TSA, SCA, HE, and XLD containing spectinomycin (5 ug/mL), novobiocin (50 ug/mL), and clindamycin (5 ug/mL). Cultures included *S.* Thompson 120, *S.* Heidelberg F5038BG1, *S.* Hadar MF60404, *S.* Enteritidis H3527, and *S.* Typhimurium H3380. Data are presented as the mean of triplicate replications of cultures and their dilutions, and error bars represent the standard deviation from the mean. Within the same serotype, comparisons of means with different letters are significantly different (*p* < 0.05) as determined by one-way ANOVA using the Holm–Sidak test for pairwise multiple comparisons; means with the same letter are not significantly different (*p* > 0.05).

Individual *Salmonella* serovars (in triplicate) were plated on four selective media containing three antibiotics after fresh growth (18 hrs, 37 °C) in TSB (1% glucose) (Figure 6). No significant differences were observed between counts on TSA or SCA, whereas three of five serovars showed slightly lower, but no significant differences when enumerated on HE, and one of five serovars showed significantly lower counts on XLD of ~1.4-log lower levels (Figure 6A). When the same triplicate dilution tube series (in 0.1% BPW) was held at refrigeration temperature (4 °C) and plated again after 3- and 6-weeks, significant differences were observed for platings on HE and XLD which showed 2–3 log lower counts relative to platings on TSA and SCA (Figure 6B,C).

### 3.7. Salmonella Stress Conditions: Exposure to Acidic Antimicrobials

**Figure 7:**
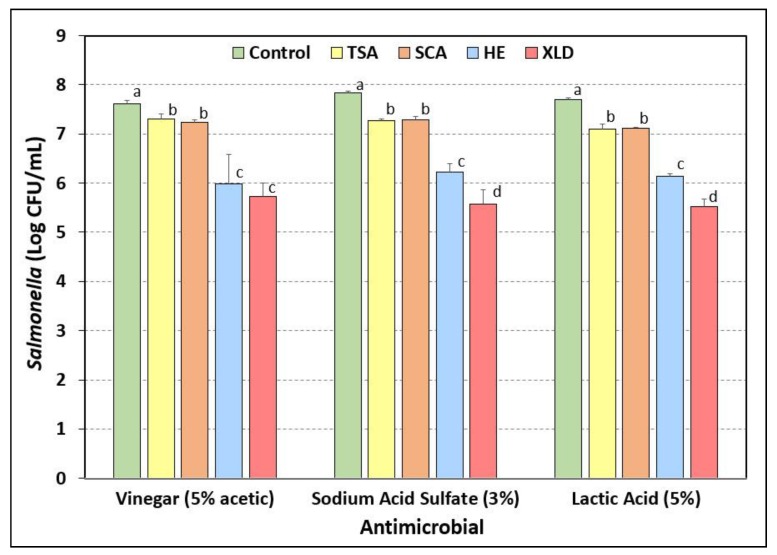
Acid-stress response of five-serovar mixtures of *Salmonella* inoculated on the surface of raw beef and subject to 30-sec dip treatment in vinegar (5% acetic acid), sodium acid sulfate (3%), and lactic acid (5%). Treated samples were then plated on TSA, SCA, HE, and XLD containing spectinomycin (5 ug/mL), novobiocin (50 ug/mL), and clindamycin (5 ug/mL). Cultures in the mixture included *S*. Thompson 120, *S*. Heidelberg F5038BG1, *S*. Hadar MF60404, *S*. Enteritidis H3527, and *S*. Typhimurium H3380. Data are presented as the mean of triplicate replications and error bars represent the standard deviation from the mean. Control represents inoculum level on beef pieces before antimicrobial treatment. Means with different letters are significantly different (*p* < 0.05) as determined by one-way ANOVA using the Holm–Sidak test for pairwise multiple comparisons to determine significant differences; means with the same letter are not significantly different (*p* > 0.05).

Beef pieces inoculated with a five-serovar mixture of *Salmonella* were subjected to 30-sec dip treatment in vinegar (5% acetic acid), sodium acid sulfate (3%), or lactic acid (5%). The beef pieces were stomached in neutralizing BPW, further diluted in 0.1% BPW, and plated onto each of four different selective media described above: TSA, SCA, HE, and XLD, all containing three antibiotics (spectinomycin, 5 ug/mL; novobiocin, 50 ug/mL; clindamycin, 5 ug/mL). Nearly the same results were obtained within each antimicrobial dip treatment: No significant differences (*p* > 0.05) were observed between TSA and SCA, which showed a modest 0.3–0.6-log reduction from the controls (Figure 7). However, enumeration on HE and XLD agars showed approximately 1.5–2.0 log lower counts than that obtained using the same dilutions for samples plated on TSA and SCA.

### 3.8. Salmonella Stress Conditions: Desiccation and Drying

**Figure 8:**
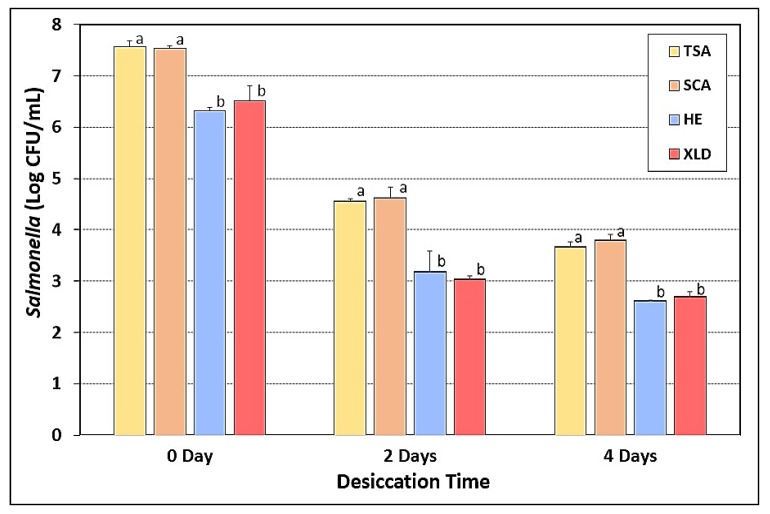
Desiccation-stress response of five-serovar mixtures of *Salmonella* inoculated on the surface of raw beef pieces and subjected up to 4-days drying in a humidity chamber at 23.9 °C (75 °F) and 55% RH. Treated samples were then plated on TSA, SCA, HE, and XLD containing spectinomycin (5 ug/mL), novobiocin (50 ug/mL), and clindamycin (5 ug/mL). Cultures in the mixture included *S*. Thompson 120, *S*. Heidelberg F5038BG1, *S*. Hadar MF60404, *S*. Enteritidis H3527, and *S*. Typhimurium H3380. Data are presented as the mean of triplicate replications and error bars represent the standard deviation from the mean. Means with different letters are significantly different (*p* < 0.05) as determined by one-way ANOVA using the Holm–Sidak test for pairwise multiple comparisons to determine significant differences; means with the same letter are not significantly different (*p* > 0.05).

Beef pieces inoculated with a five-serovar mixture of *Salmonella* were dry-marinaded with biltong spices and salt (no vinegar) before hanging in a humidity oven to be subjected to drying for up to 4 days at 75 °F and 55% RH. Samples retrieved at 0-, 2-, and 4-days of drying were plated on the four selective media described earlier (TSA, SCA, HE, and XLD, all containing three antibiotics). Again, the TSA and SCA based media had nearly identical counts, while HE and XLD based media both showed 1–1.5-log lower counts than the other selective media at each assay time (Figure 8).

### 3.9. Salmonella Stress Conditions: Thermal Stress

**Figure 9:**
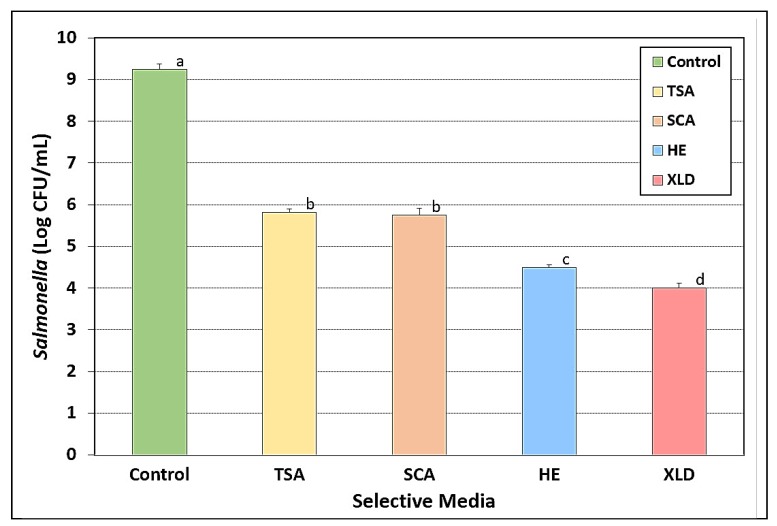
Thermal-stress response of five-serovar mixture of *Salmonella* heated at 62.8 °C (145 °F) for 75 sec. Treated samples were then plated on TSA, SCA, HE, and XLD; all contained spectinomycin (5 ug/mL), novobiocin (50 ug/mL), and clindamycin (5 ug/mL). Cultures in the mixture included *S.* Thompson 120, *S.* Heidelberg F5038BG1, *S.* Hadar MF60404, *S.* Enteritidis H3527, and *S.* Typhimurium H3380. The control is the level of the mixed culture before heating. Data are presented as triplicate replications and error bars represent standard deviation from the mean. Means with different letters are significantly different (*p* < 0.05) as determined by one-way ANOVA using the Holm–Sidak test for pairwise multiple comparisons to determine significant differences; means with the same letter are not significantly different (*p* > 0.05).

When 1-mL samples of the combined *Salmonella* serovars were subjected to heating at 145 °F (62.8 °C) for 75 sec in thin layer bags, equivalent counts were obtained on both TSA and SCA agar (~3.4–3.5-log reduction). Counts retrieved on HE and XLD agars showed microbial reductions of 4.75-log and 5.25-log, respectively (Figure 9).

### 3.10. Bacterial Injury as Determined by Plating on Selective vs. Nonselective Media

When microbial counts of acid-adapted Salmonella were plated on TSA, and compared to platings on SCA, XLD, and HE, reduced levels of recovered counts were consistently obtained on XLD and HE (Table 2). This is consistent with the inhibition of sublethally injured cells by selective media components, as observed by Wesche et al. [32]. As determined with XLD and HE agars, injured cells were determined to comprise as much as 14%–19% of fresh acid-adapted cells and 73.0%–99.7% of the microbial populations of the remaining 11 trials after stressed conditions (Table 2). A similar comparison of acid-adapted *Salmonella* plated on SCA with those plated on TSA demonstrated a modest 4.4%–13.7% injury level in four of twelve trials, while showing 1.9%–46.7% enhancement of microbial recovery in eight of twelve trials (Table 2).

## 4. Discussion

In this study, various *Salmonella* serovars (*S.* Thompson 120, *S.* Heidelberg F5038BG1, *S.* Hadar MF60404, *S.* Enteritidis H3527, S. Typhimurium H3380) were characterized prior to their use as inocula in subsequent studies to evaluate antimicrobial interventions applied during the processing of dried beef (i.e., biltong, beef jerky). The *Salmonella* strains used in this study are widely distributed in academia and government research labs and have a long history of testing on the effects of antimicrobial interventions against *Salmonella* applied to meat and poultry products [22,23,24,25,32,38,39,40,41,42].

Characterization of antibiotic resistance demonstrated that they are not only multi-drug resistant but are also resistant to the same antibiotics. This enables the use of the common antibiotics for the preparation of selective media to recover them from inoculated food studies. TSA containing novobiocin, spectinomycin, and clindamycin was considered a useful generic selective agar media for these *Salmonella* serovars. The approach to use antibiotics to which the inoculum bacteria are resistant has long been an effective method of selective enumeration of inoculum strains recovered from non-sterile foods that also contain other microorganisms [4,5,6,7,8,9]. However, the appearance of high levels of multi-drug resistant background bacteria from un-inoculated raw beef on TSA containing the three antibiotics was cause for concern and precipitated a search for a more selective medium.

The intended use of the *Salmonella* serovars was as inocula prior to antimicrobial (acid) interventions on raw beef, and therefore, the strains were acid-adapted prior to use throughout this study. Acid adaptation of *Salmonella* serovars was reported in the early 1990s [43,44,45] whereby *Salmonella* pre-exposed to low pH were more resistant to acidic conditions than non-adapted cells. Foster and others [43,45] adjusted media with HCl to achieve low pH conditions. Subsequently, during investigations with enterohemorrhagic *Escherichia coli* and *Listeria monocytogenes*, Buchanan et al. [46,47] augmented media with glucose to allow the organisms to lower pH during growth to induce the acid adaptation response. The ability of *Salmonella* and Shigatoxigenic *E. coli* to adapt to stressful environments has significant implications in the safety of processed foods [43,48,49]. The *Salmonella* cultures used throughout this study were predisposed to acidic stress conditions by growth in media containing 1% glucose. This has been recommended by the NACMCF [9] for inoculated challenge studies and by USDA-FSIS when evaluating antimicrobial food processes involving acidic treatments.

Sodium pyruvate was initially considered as a possible media additive to allow recovery of injured cells since many intended applications would be stress-related. However, when examined in the context of nutrient depleted/starved cells, or when *Salmonella*-inoculated beef was exposed to the stress of salt (dehydration) and vinegar (acid) marination, no benefit was observed with sodium pyruvate. Similarly, neither Knabel and Thielen [50] nor Kirby and Davies [51] observed improved recovery of heat-injured *Listeria* or *Salmonella*, respectively, when using sodium pyruvate in their recovery medium. Various investigators have shown that acid adaptation plays a role in enhanced survival of *Salmonella* and other bacteria, offering cross-protection not only against acid stress, but desiccation, salt, and thermal stress as well [44,52,53,54]. Although other injury recovery additives may have been more effective than sodium pyruvate (i.e., catalase, yeast extract), we continued without additional additives, since the SCA medium was demonstrating comparable results to TSA. In our study, enumeration of *Salmonella* was consistently and significantly lower on XLD and HE agars, as they likely inhibited the recovery of injured cells [10,11,55]. XLD or HE would have solved our background microorganism problem as the *Salmonella* would have appeared as black colonies. However, our data showed lower counts (corresponding to injury levels of 89%–99%) when *Salmonella* were plated on XLD or HE and compared to levels recovered on TSA. Strantz and Zottola [55] were only able to improve recovery with XLD and HE media by plating cells on TSA, allowing them to sit for 4 hrs of recuperation, and then overlaying with XLD or HE agar. Subsequent work by various investigators led to several modifications of the selective overlay technique, including the agar underlay [21] and thin agar layer [19,20,56,57] methods.

## 5. Conclusions

The *Salmonella* serovars examined in this study were intended for use in USDA-FSIS validation studies on antimicrobial interventions for dried beef processing to achieve 5-log reduction of *Salmonella*. Hence, it was important to accurately enumerate *Salmonella* survivors during processing in lieu of potential background bacteria from raw meat. Selenite cystine broth, a medium routinely used as a selective enrichment broth for luxuriant growth of *Salmonella* spp., provided more suppression of background microorganisms from raw beef when used as an agar medium than did TSA (with antibiotics). The ability to quantitatively enumerate these *Salmonella* serovars on SCA containing antibiotics facilitated experimental procedures and eliminated the need for cumbersome multiple layered plating schemes. Enumeration with SCA was not significantly different than that obtained with TSA, which demonstrates the adequacy of SCA as a selective media for these *Salmonella* serovars, and more so when supplemented with antibiotics to which the strains are resistant. The use of SCA should allow quantitative enumeration of these acid adapted *Salmonella* serovars when recovered from inoculated food studies employing antimicrobial interventions in spite of the background organisms that might be present.

## Figures and Tables

**Table 1:** Antibiotic disc assay of select Salmonella serovars showing multidrug resistance (Res, resistant; Sens, sensitive; S-sens, slightly sensitive; V-sens, very sensitive).

*Salmonella* Serovars	Clin	Novo	Oxa	Pen	Van	Spec	Amo	Amp	Pip	Str
	CC 2*	NB 5	OX 1	P 10	VA 30	10 ug	AMC 30	AM 10	PIP 100	S 10
***S*. Enteritidis H3527**	Res	Res	Res	Res	Res	Res	Sens	Sens	Sens	Sens
***S*. Hadar MF60404**	Res	Res	Res	Res	Res	Res	Res	Res	Res	Res
***S*. Heidelberg F5038BG1**	Res	Res	Res	Res	Res	Res	Res	Res	Res	Res
***S*. Montevideo FSIS 051**	Res	Res	Res	Res	Res	Res	Sens	S-Sens	V-Sens	S-Sens
***S*. Thompson 120**	Res	Res	Res	Res	Res	Res	Res	Res	Res	Res
***S*. Typhimurium H3380**	Res	Res	Res	Res	Res	Res	Res	Res	Res	Res

Antibiotics: Clin (Clindamycin), Novo (Novobiocin), Oxa (Oxacillin), Pen(Penicillin), Van (Vancomycin), Spec (Spectinomycin), Amo (Amoxicillin), Amp (Ampicillin), Pip (Piperacillin), Str (Streptomycin). * BD BBL Sensi-Disc product designations.

**Table 2:** Determination of % injury (-%) or enhancement (+%) of *Salmonella* by comparison of microbial counts on selective media (SCA, XLD, HE) to non-selective meda (TSA).

Process	% Injury (-) or %Enhancement (+)		
	SCA	XLD	HE
**Sodium pyruvate trials** Nutrient depletion/starvation Acid/vinegar marinade	+46.5 −13.7	−94.0 −99.7	−94.9 −98.5
**Extended nutrient depletion/starvation** Fresh cells 3-weeks 6-weeks	+1.93 +16.1 +6.6	−14.4 −99.4 −98.6	−19.0 −99.7 −99.2
**Acidic stress (inoculated beef, 30-sec dip)** Vinegar (5% acetic acid) Lactic acid (5%) Sodium acid sulfate (3%)	−4.4 +4.8 +2.7	−95.1 −97.3 −97.9	−96.5 −89.1 −73.0
**Desiccation stress (salt/spiced beef, dried)** 0-days 2-days 4-days	−6.5 +17.6 +31.8	−91.1 −96.9 −89.3	−94.4 −95.8 −91.1
**Thermal stress** (62.8 °C/145 °F for 75 sec)	−10.6	−99.2	−99.6
